# BMP-7 Enhances the Angiogenic Potential of Wharton’s Jelly Mesenchymal Stem Cells Seeded in Decellularized Human Dental Pulp

**DOI:** 10.3390/ijms27198599

**Published:** 2026-09-25

**Authors:** Wei Ying Peh, Dalia Abdullah, Sook Luan Ng, Eason Soo, Nurul Inaas Mahamad Apandi, Nur Azurah Abdul Ghani

**Affiliations:** 1 Department of Restorative Dentistry, Faculty of Dentistry, Universiti Kebangsaan Malaysia, Kuala Lumpur 50300, Malaysia; p138662@siswa.ukm.edu.my (W.Y.P.); easonso@ukm.edu.my (E.S.); 2 Department of Craniofacial Diagnostics and Biosciences, Faculty of Dentistry, Universiti Kebangsaan Malaysia, Kuala Lumpur 50300, Malaysia; ngsookluan@ukm.edu.my (S.L.N.); nurulinaas@ukm.edu.my (N.I.M.A.); 3 Department of Obstetrics and Gynecology, Faculty of Medicine, Universiti Kebangsaan Malaysia, Kuala Lumpur 56000, Malaysia; azurah@ukm.edu.my

**Keywords:** biocompatibility, degradation rate, regenerative endodontics, swelling ratio, scaffold pore size, VEGF-A

## Abstract

Regeneration of a pulp–dentin complex requires coordinated angiogenesis. This study investigated the angiogenic potential of Wharton’s jelly mesenchymal stem cells (WJMSCs) in decellularized human dental pulp (DHDP) treated with bone morphogenetic protein-7 (BMP-7). Decellularized human dental pulp tissues were repopulated with WJMSCs, with or without BMP-7 (50 ng/mL). Degradation rate, swelling ratio, and pore size of DHDP samples were assessed. DHDP and repopulated DHDP were evaluated through hematoxylin and eosin and 4′, 6-diamidino-2-phenylindole staining, and field-emission scanning electron microscopy. Biocompatibility of DHDP, BMP-7, and DHDP+BMP-7 on WJMSCs was assessed using the MTT assay. WJMSCs’ viability was evaluated using a resazurin-based assay on days 7, 14, and 21. Angiogenic potential was assessed by measuring vascular endothelial growth factor-A (*VEGFA*) gene expression via quantitative reverse transcriptase polymerase chain reaction and VEGF-A protein expression via immunohistochemistry. At 21 days, DHDP exhibited a degradation rate of 40.19%, moderate swelling (77.22 ± 0.59%), and a significantly larger mean pore size (12.393 ± 0.638 µm^2^) than HDP (5.956 ± 0.501 µm^2^; *p* = 0.039). Histologically, DHDP exhibited complete nuclei removal, while WJMSCs were well integrated into the DHDP stroma. DHDP, BMP-7, and DHDP+BMP-7 were non-cytotoxic to WJMSCs. The DHDP+WJMSC+BMP-7 group demonstrated significantly higher cell viability at all time points compared with DHDP+WJMSC alone (*p* < 0.01), and *VEGFA* expression was significantly increased at both mRNA (*p* = 0.002) and protein levels. BMP-7 (50 ng/mL) can enhance WJMSC proliferation and *VEGFA*/VEGF-A expression within DHDP scaffolds while maintaining biocompatibility.

## 1. Introduction

Root canal treatment (RCT) is one of the most common endodontic procedures for managing pulp and periapical diseases. Despite its favorable success rate of 82.8–89.1% [1], RCT removes the pulp and therefore cannot preserve or restore its neurovascular, sensory, and immunological functions [2]. Regenerative endodontic therapy (RET) offers an alternative approach to physiologically replace the tissues and cells of the pulp–dentin complex. The current accepted procedure involves disinfecting the root canal and recruiting endogenous cells into the canal space, usually via an induced blood clot [3]. In necrotic immature permanent teeth, a systematic review and meta-analysis by Ong et al. reported high survival, healing, and continued root development following RET [4]. However, a systematic review of human histological assessments revealed that the tissues formed after cell-homing RET are predominantly reparative [5]. These tissues commonly comprise fibrous connective tissue, cementum-like tissue, bone-like tissue, and periodontal ligament-like tissue. Mixed features of repair and regeneration have been reported less consistently, while complete restoration of the organized native pulp–dentin complex has not been demonstrated in the available human histological studies [6].

In contrast, conflicting outcomes have been reported when RET is performed in mature teeth [7]. A systematic review by Kasper et al. reported success rates ranging from 60% to 100% [8], whereas a systematic review and meta-analysis by Scelza et al. found only moderately positive responses to pulp sensitivity tests, with very low certainty of evidence [9]. The outcome of cell-homing procedures depends in part on the availability and viability of recruited endogenous stem cells, which may be compromised by excessive chemical disinfection and instrumentation beyond the apex, thereby inducing intracanal bleeding [10]. The variability in outcomes observed in mature teeth may also be related to the reduced size of the apical papilla and the lower viability of apical papilla cells in mature roots [11,12]. Furthermore, disinfection and instrumentation beyond the apical foramen may provoke an inflammatory response and the formation of an inflammatory apical plug, potentially limiting the migration of periapical cells into the root canal and thereby impairing pulp tissue regeneration [13]. Histological assessments have supported this finding by demonstrating inflammatory cell infiltration after RET [14,15,16].

These limitations have encouraged the development of cell-based pulp tissue engineering, in which selected stem cells are introduced together with a three-dimensional scaffold and bioactive molecules that regulate cell survival, migration, and differentiation [17]. True pulp regeneration requires more than the formation of vascularized connective tissue within the canal. The native dental pulp is an organized, vascularized, and innervated connective tissue that supports odontoblast activity, dentin homeostasis, nutrition, immune defense, and sensory function [18]. Functional pulp–dentin regeneration therefore requires coordinated vascular ingrowth and neural integration [19,20]. Angiogenesis is a crucial prerequisite in pulp regeneration. The implanted cells within a three-dimensional scaffold are vulnerable to hypoxia and nutrient deprivation before connection with the host circulation; hence, they initially depend on vascular supply for oxygen and nutrient delivery [21]. A recent review suggested that angiogenic and neurogenic regeneration remain major barriers to the large-scale clinical application of pulp tissue engineering [19]. Contemporary regenerative models therefore increasingly attempt to coordinate vascular and neural responses rather than assessing a single biological outcome.

A clinical study using transplanted autologous human deciduous pulp stem cells demonstrated de novo regeneration of functional pulp tissue, with histological evidence of vascularized, innervated pulp-like tissue formation, thereby supporting the feasibility of cell-based pulp regeneration [22]. This regenerative potential may be related to the high proliferative capacity and multilineage differentiation potential of stem cells derived from human deciduous teeth. However, the routine clinical use of autologous deciduous dental pulp stem cells is limited by the need for an available donor tooth, particularly in adult patients. These limitations highlight the need for a more accessible and clinically translatable cell source within the tissue-engineering triad of stem cells, bioactive signaling molecules, and scaffolds. Despite continued advances in pulp tissue engineering, no single biomaterial or cell–scaffold–signaling molecule has yet emerged as the preferred combination for predictable and functional pulp–dentin regeneration [23,24].

Among potential stem cell sources for pulp tissue engineering, Wharton’s jelly mesenchymal stem cells (WJMSCs) are increasingly recognized for their regenerative potential due to their multilineage differentiation potential, high proliferative capacity, and immunomodulatory properties [25]. WJMSCs can differentiate into multiple cell lineages, including odontoblasts that contribute to dentin formation, highlighting their potential for regenerative endodontic applications [26]. Compared with dental stem cells, WJMSCs can be obtained non-invasively and readily from discarded umbilical cord tissue with maternal consent, making them a more accessible and ethically acceptable cell source [27]. Additionally, WJMSCs exhibit low immunogenicity [28,29] and low tumorigenic potential [30], supporting their potential safety for regenerative applications [31]. Their capacity for large-scale expansion and cryopreservation further supports the future development of a standardized allogeneic cell source. WJMSCs have also demonstrated their ability to promote blood vessel formation in both in vitro and in vivo dermal regeneration via high expression of pro-angiogenic molecules [32]. However, the cellular behavior of WJMSCs can be influenced by the surrounding extracellular matrix. Consequently, findings obtained from non-dental tissues or conventional culture systems cannot be directly extrapolated to a tissue-specific dental pulp microenvironment. In particular, the angiogenic behavior of WJMSCs following recellularization within a decellularized human dental pulp scaffold remains largely unexplored.

Decellularized human dental pulp (DHDP) is a tissue-specific extracellular matrix scaffold obtained by removing cellular and nuclear content while preserving the native pulp extracellular matrix. Unlike conventional natural or synthetic scaffolds, DHDP retains the native pulp architecture, matrix composition, and cell-binding environment that influence cell attachment and migration [33]. This tissue specificity is clinically relevant because the extracellular matrix with retained biochemical cues and its three-dimensional organization [34] may reproduce biological cues absent in non-pulp-derived biomaterials. Recent experimental evidence further supports the importance of tissue-specific extracellular matrices in pulp regeneration. Liang et al. demonstrated that a dental pulp-derived decellularized matrix hydrogel promoted enhanced odontogenic differentiation of dental pulp stem cells and enhanced pulp-like tissue formation compared with a decellularized nerve-derived matrix hydrogel, highlighting the advantages of using a pulp-specific scaffold [35].

The decellularization process also reduces the risk of disease transmission [36] and minimizes antigenicity of the scaffold [33], making allogeneic transplantation for pulp regeneration feasible. Although DHDP scaffolds have been shown to support cell attachment and proliferation, decellularization may remove or alter matrix-bound growth factors that influence cellular behaviors [37]. A systematic review found favorable preclinical cellular and tissue responses of decellularized dental pulp scaffolds but identified substantial methodological heterogeneity and insufficient evidence to determine their overall efficacy [38]. Furthermore, most recent studies on dental pulp-derived extracellular matrices have focused on solubilized or injectable hydrogels rather than intact decellularized human pulp scaffolds [35]. Consequently, the biological behavior and physical characteristics of an intact decellularized DHDP scaffold require further investigation, particularly following recellularization with stem cells and supplementation with pro-angiogenic signaling molecules.

Bone morphogenetic protein 7 (BMP-7) is known for its essential roles in the osteogenic [39] and dentinogenic [40] potential of mesenchymal stem cells in pulp regeneration. BMP-7 has been reported to participate in the maturation phase of angiogenesis by promoting endothelial cell proliferation and migration, as well as the migration and differentiation of vascular smooth muscle cells through signaling pathways involving anaplastic lymphoma kinase receptors (ALK2, ALK3, and ALK6) [41]. Previous studies have investigated the pro-angiogenic effect of BMP-7 on endothelial cells [42] and other stem cells [43]. These findings suggest that BMP-7 may contribute to a multifunctional regenerative microenvironment by supporting osteogenic, dentinogenic, and pro-angiogenic signaling. However, its angiogenic influence on WJMSCs remains largely unexplored. Hence, the present study aims to evaluate the modulatory effect of BMP-7 on the angiogenic potential of WJMSCs repopulated within DHDP scaffolds.

The combination of WJMSCs, DHDP, and BMP-7 is therefore biologically relevant because it integrates a bankable multipotent cell source, a tissue-specific human pulp matrix, and a multifunctional growth factor capable of regulating regenerative processes. Recently, our research group demonstrated that BMP-7 promoted the dentinogenic differentiation of WJMSCs repopulated into DHDP [40]. However, whether BMP-7 enhances the pro-angiogenic potential of WJMSCs within the same tissue-specific scaffold remains insufficiently characterized. Addressing this knowledge gap is important because successful full-thickness pulp regeneration requires adequate vascular integration to maintain the viability of regenerated pulp tissue. Therefore, this study characterized the physical and morphological properties of DHDP, evaluated the biocompatibility of DHDP and BMP-7, and investigated the effects of BMP-7 on the viability, *VEGFA* gene, and protein expression of WJMSCs repopulated within DHDP. It was hypothesized that BMP-7 would enhance WJMSC viability and *VEGFA* gene and protein expression without compromising the biocompatibility of the tissue construct.

## 2. Results

### 2.1. Characterization of WJMSCs

WJMSCs were successfully isolated from human umbilical cord tissue and expanded up to passage 4. Upon examination using an inverted light microscope, the adherent cells displayed a uniform, spindle-shaped, fibroblast-like morphology characteristic of MSCs (Figure 1a). The cells maintained high viability and consistent morphology throughout successive passages.

The WJMSCs’ stemness and mesenchymal stem cell phenotype were confirmed by the positive expression of NANOG, OCT4, SOX2, CD73, CD90, CD105, and negligible expression of CD34, CD45, and HLA-DR (Table 1). Trilineage differentiation assays (adipogenic, osteogenic, and chondrogenic) further confirmed their multipotency (Figure 1b–d).

### 2.2. Characterization of DHDP

The hematoxylin and eosin (H&E) samples of the untreated pulp tissue showed densely packed nuclei stained in dark purple, indicating an abundance of native dental pulp cells. The fibrous ECM was interspersed among these cellular components, with a higher cellular density along the periphery (Figure 2a). The endothelial cells can be seen lining the vessels.

On the other hand, the DHDP samples showed complete removal of cellular content, as indicated by the absence of hematoxylin-positive nuclear staining. Despite decellularization, the ECM structure remained intact, with an open, porous appearance, and maintained its structural integrity, suggesting the retention of collagenous and non-cellular ECM components essential for future cellular repopulation (Figure 2b).

In 4′,6-diamidino-2-phenylindole (DAPI)-stained, untreated pulp tissue samples, a dense, uniform nuclear distribution was observed, with numerous fluorescent blue nuclear signals throughout the tissue matrix, indicating high, well-organized cellularity. The parallel alignment of nuclei matched the native stromal arrangement of dental pulp cells (Figure 3a). In contrast, the DHDP sample showed no DAPI-positive nuclei, as indicated by a completely dark field with no nuclear fluorescence visible throughout the scaffold section, signifying successful removal of cellular components (Figure 3b).

### 2.3. Morphological Features of DHDP

In field emission scanning electron microscopy (FESEM) images, the fibrous ECM of the untreated pulp tissue samples appeared densely interwoven and was interspersed with numerous globular and particulate structures suggestive of cellular content. These features were consistent with the presence of intact dental pulp cells, confirming the native cellular composition of the pulp tissue (Figure 4a). The DHDP samples demonstrated a markedly different histoarchitecture. The DHDP exhibited a well-preserved, extensive collagen bundle but a more exposed and porous fibrous network. Notably, the DHDP lacked discernible cellular content, indicating successful decellularization (Figure 4b).

### 2.4. Pore Size Measurement of DHDP

DHDP showed a larger average pore size (12.393 ± 0.638 µm^2^), which was significantly greater (*p* = 0.039) than that of human dental pulp (5.956 ± 0.501 µm^2^) (Figure 5). After repopulation with WJMSCs, the average pore size decreased to 8.324 ± 0.466 µm^2^ in the control group (DHDP+WJMSC), while it further decreased to 6.219 ± 0.450 µm^2^ in the experimental group (DHDP+WJMSC+BMP-7) (Figure 5). These results suggest that decellularization increases scaffold porosity, and subsequent cell seeding causes partial pore filling, indicating successful repopulation.

Standardization and calibration were performed prior to pore size measurement. The results demonstrated substantial agreement, with the inter-examiner Cohen’s kappa coefficient of 0.77. The intra-examiner Cohen’s kappa coefficient after a 4-week interval was also 0.77, thereby validating the reliability of the measurement process.

### 2.5. Physical Properties of DHDP

#### 2.5.1. Water Absorption

The hydrophilic property of DHDP, which is essential for nutrient diffusion, cellular infiltration, and migration, was evaluated by measuring its swelling behavior. After a 24-h immersion in 1× PBS, the DHDP exhibited a swelling ratio of 77.22 ± 0.59%, indicating an acceptable capacity for fluid absorption.

#### 2.5.2. Degradation Rate

The degradation profile of DHDP was tracked over 21 days in 1× PBS to assess the scaffold’s structural stability. The average degradation percentages on days 7, 14, and 21 were 20.27 ± 0.44%, 30.83 ± 0.57%, and 40.19 ± 0.22%, respectively, with a significant difference observed between day 7 and day 21 (*p* = 0.002) (Figure 6). These findings demonstrate a gradual, time-dependent degradation pattern that aligns with ongoing scaffold resorption in a regenerative environment, thereby supporting cell repopulation.

### 2.6. Biocompatibility of DHDP, BMP-7, DHDP+BMP-7

During the optimization of the BMP-7 concentration stage, 50 ng/mL BMP-7 was found to significantly enhance cell viability to 115.54 ± 0.53% (*p* = 0.002), showing the greatest increase in proliferation compared to the control group (0 ng/mL BMP-7, defined as 100%) (Figure 7a). A decrease in viability was observed at higher concentrations, indicating a potential cytotoxic effect of high-concentration BMP-7.

### 2.7. Cytotoxicity Test of DHDP, BMP-7, DHDP+BMP-7 on WJMSCs

None of the DHDP, BMP-7, or DHDP+BMP-7 groups exhibited cytotoxicity toward WJMSCs, as all groups had cell viability percentages above 70%. However, viability was lower than that of the positive control group (DMEM + 10% FBS) (Figure 7b).

### 2.8. Viability of WJMSCs Repopulated into DHDP

In the DHDP+WJMSC group, cell viability showed a gradual increase from day 7 (20.73 ± 0.75%) to day 14 (22.34 ± 0.93%), reaching 25.67 ± 0.59% by day 21 (Figure 7c). In comparison, the DHDP+WJMSC+BMP-7 exhibited consistently higher viability across all time points, with mean values of 30.16 ± 0.52%, 31.86 ± 0.80%, and 32.47 ± 0.32% on day 7, 14, and 21, respectively. There was a significantly higher viability in the DHDP+WJMSC+BMP-7 compared with DHDP+WJMSC on day 7 (*p* = 0.002), day 14 (*p* < 0.01), and day 21 (*p* < 0.01), indicating a consistent enhancement in WJMSC viability with BMP-7 treatment.

Within-group comparisons further revealed a significant increase in viability in the DHDP+WJMSC group from day 7 to day 21 (*p* = 0.007). Likewise, the DHDP+WJMSC+BMP-7 group showed a significant increase from day 7 to day 21 (*p* = 0.033) (Figure 7c). These findings support the proliferative effect of BMP-7 on WJMSCs repopulated within DHDP scaffolds over time.

### 2.9. Histological Analysis of DHDP Repopulated with WJMSCs

In the repopulated DHDP samples at 21 days, cellular repopulation was observed within the scaffold. However, cells were predominantly distributed toward the periphery of the DHDP, with limited cellular infiltration into deeper scaffold regions (Figure 8a). A similar appearance was observed in the experimental group (DHDP+WJMSC+BMP-7) (Figure 8b).

### 2.10. Angiogenic Expression of Repopulated DHDP

The relative mRNA expression level of *VEGFA* in DHDP+WJMSC+BMP-7 (0.1045 ± 0.0048) was significantly higher than that of DHDP+WJMSC (0.0474 ± 0.0041) (*p* = 0.002), indicating a significant upregulation in angiogenic signaling (Figure 7d).

In untreated pulp tissues, intense VEGF-A immunoreactivity was observed by immunohistochemistry (IHC), characterized by strong brown chromogenic staining throughout the section (Figure 9a). This indicates active VEGF-A protein expression and positive angiogenic signaling. As expected, no VEGF-A staining was detected in the acellular DHDP scaffolds, confirming the absence of endogenous cellular activity and angiogenic signaling (Figure 9b). Among the repopulated groups, weak, diffuse brown staining was observed in the DHDP+WJMSC group, primarily at the scaffold periphery, suggesting low VEGF-A expression (Figure 9c). In contrast, the DHDP+WJMSC+BMP-7 group exhibited a notable increase in VEGF-A immunopositivity compared with DHDP+WJMSC, with more intense and widespread brown cytoplasmic staining in cells distributed throughout the scaffold (Figure 9d). This enhanced expression suggests that BMP-7 treatment increases VEGF-A-associated pro-angiogenic signaling in WJMSCs, potentially supporting neovascularization within the engineered tissue construct.

## 3. Discussion

The present study demonstrated that the pulp construct comprising DHDP, WJMSCs, and BMP-7 provides a biologically favorable microenvironment that supports stem cell survival and enhances *VEGFA* gene and protein expression. The DHDP scaffold retained its native extracellular matrix architecture after decellularization. It exhibited suitable physical properties, including gradual degradation, moderate swelling, and increased pore size, all of which are important for scaffold remodeling and nutrient exchange. Furthermore, DHDP was biocompatible toward WJMSCs and supported sustained cell viability throughout the culture period. Supplementation with BMP-7 further enhanced WJMSC proliferation and significantly increased *VEGFA* gene and protein expression. Collectively, these findings suggest that BMP-7 not only maintains cellular viability within a tissue-specific extracellular matrix but also promotes a biological environment favorable for future vascularization, an essential prerequisite for functional pulp regeneration rather than merely reparative tissue formation.

Successful regeneration of complex tissues depends not only on the presence of viable stem cells and bioactive signaling molecules but also on an extracellular matrix capable of recreating the native biological niche. In the present study, histological and FESEM analyses confirmed complete removal of cellular components while preserving the three-dimensional extracellular matrix architecture of human dental pulp. Preservation of this tissue-specific matrix is particularly important because the extracellular matrix functions as an instructive microenvironment rather than merely a structural framework. Other than providing mechanical support, extracellular matrix proteins such as collagen, fibronectin, laminin, and glycosaminoglycans regulate cell adhesion, migration, and lineage commitment through cell–matrix interactions [44,45]. ECM proteins also bind and stabilize angiogenic growth factors such as VEGF-A and PDGF-BB, which play essential roles in vascular development and maturation [46]. Recent studies increasingly support the concept that tissue-specific extracellular matrices possess biological properties as compared to conventional scaffold materials. Liang et al. demonstrated that a dental pulp-derived decellularized extracellular matrix hydrogel promoted superior odontogenic differentiation and pulp-like tissue formation compared with a decellularized nerve-derived matrix, highlighting that tissue origin influences stem-cell behavior rather than merely providing structural support. In addition, decellularized bovine dental pulp combined with dental pulp stem cells has been shown to upregulate angiogenic markers such as KDR and PECAM-1 following implantation [47]. The findings of the present study are consistent with previous studies demonstrating that DHDP preserves the natural extracellular matrix architecture that supports stem cell attachment and proliferation.

The physical characteristics of a scaffold strongly influence its performance in tissue engineering applications by affecting cellular behavior [48,49]. An ideal scaffold should degrade at a rate that matches the rate of new tissue formation, especially during the initial phase, to maintain sufficient structural integrity [50]. In the present study, the DHDP scaffold exhibited a rapid-to-moderate degradation profile over 21 days in vitro, suggesting that the scaffold may provide temporary structural support while permitting ongoing tissue remodeling. This is notably greater than the degradation rate reported in prior work using a crosslinked decellularized amniotic membrane–hydrogel scaffold at 28 days [51]. While this may facilitate early WJMSC migration and extracellular matrix deposition, premature degradation could destabilize the scaffold core and limit deeper cell infiltration [52,53]. This may explain the predominantly peripheral cellular distribution observed in the histological examinations of the present study.

Scaffold architecture, particularly porosity and pore size, also plays a crucial role in cell distribution [54]. Pore sizes larger than approximately 100 µm generally support effective cell attachment and migration [54]. However, excessively large pores may reduce the internal surface area for cell adhesion and increase scaffold permeability, thereby reducing cell retention and attachment during seeding [55,56,57]. Conversely, smaller pores may trap cells at the scaffold surface and restrict nutrient diffusion, resulting in uneven cell distribution in the scaffold’s central core [55]. As proliferation progresses and cell density increases, the effective pore radius decreases, potentially reducing nutrient flux to the inner scaffold zones [58,59]. This is consistent with our finding showing a reduction in pore size in both DHDP+WJMSC+BMP-7 and DHDP+WJMSC as compared with DHDP. In the present study, pore size was quantified as pore area rather than individual pore diameter. Despite the increased area of DHDP observed in FESEM and H&E examination compared with HDP, histological and DAPI analyses of repopulated scaffolds revealed superficial cellular attachment. This observation suggests that increasing pore size alone may not be sufficient to achieve complete three-dimensional cellular infiltration. Instead, pore interconnectivity and diffusion pathways are likely to play equally important roles in determining the efficiency of scaffold recellularization. A similar pattern was reported by Matoug-Elwerfelli et al. [60], in which cells remained localized at the periphery of a decellularized rat dental pulp despite the clear presence of porosity in histological sections. However, no quantitative measurements of pore size or porosity were performed in that study [60]. This suggests that limited cell penetration remains a common challenge in intact pulp-derived extracellular matrices rather than being unique to the present scaffold design.

Scaffolds with high swelling capacity are often advantageous for enhancing nutrient and culture media diffusion and absorption. However, a scaffold with an excessive swelling ratio can diminish its elasticity, resulting in transient pore collapse due to increased pore size and porosity [51], ultimately impairing mechanical integrity [61]. In this study, the swelling ratio of the DHDP indicated moderate-to-non-swelling behavior [62], which facilitates nutrient diffusion without compromising scaffold stability [59]. The present findings therefore indicate that the physical characteristics of DHDP are compatible with the biological requirements of pulp tissue engineering.

Biocompatibility is a fundamental requirement for clinical translation of biomaterials [63]. In this study, DHDP leachate, 50 ng/mL BMP-7, and their combination demonstrated no cytotoxic effects on WJMSCs. Although a slight reduction in metabolic activity was observed compared with untreated controls, the difference was not statistically significant. This reduction may be attributed to residual decellularization agents [37,64] and to possible oxidative stress caused by degradation byproducts [65]. Degradation-related oxidative stress may activate redox-sensitive signaling pathways, such as mitogen-activated protein kinase (MAPK) and c-Jun N-terminal kinase (JNK) pathways, which can transiently reduce mitochondrial activity without inducing apoptosis [66]. Consequently, metabolic assays such as MTT or Alamar Blue may detect reduced metabolic output despite preserved cell viability [67].

In the present study, BMP-7 treatment significantly enhanced WJMSC proliferation, further confirming its non-cytotoxic effect. The optimal concentration identified in this study was 50 ng/mL, which produced the highest metabolic activity in dose–response experiments. Similar findings have been reported in dental pulp stem cells, where 50 ng/mL BMP-7 promoted proliferation and angiogenic activity [43,68]. Conversely, Kim et al. [69] reported that higher doses of BMP-7 (75–100 ng/mL) facilitated enhanced mineralization while also reducing proliferation rates. This was explained by the stimulation of early differentiation through G1-phase arrest [70] or by potential feedback-inhibitory mechanisms involving upregulation of BMP antagonists such as noggin or gremlin [69].

A major finding of this study was the enhanced proliferation and angiogenic potential of WJMSCs in the DHDP scaffold following BMP-7 treatment. Rather than functioning solely as an osteogenic or dentinogenic morphogen, BMP-7 appears to influence WJMSC survival and VEGF-associated pro-angiogenic expression, both of which are critical during the early stages of tissue regeneration when transplanted cells remain dependent on diffusion for oxygen and nutrient supply. Although the present study did not directly evaluate endothelial cell behavior or neovascularization, the increased VEGF-A expression observed in BMP-7-treated constructs suggests an enhanced pro-angiogenic potential that may facilitate subsequent vascular integration following implantation.

VEGF-A is one of the principal regulators of angiogenesis, stimulating endothelial cell proliferation, migration, and vascular permeability through activation of VEGFR1 and VEGFR2 [46,71,72]. In the context of pulp regeneration, rapid vascularization is particularly important because the regenerated tissue is confined within rigid dentinal walls and receives its blood supply exclusively through the narrow apical foramen. Consequently, delayed vascular ingrowth may result in hypoxia, nutrient deprivation, and subsequent loss of transplanted cells. Recent reviews have identified early vascularization as one of the major determinants of successful pulp regeneration rather than merely pulp repair [19,20,21].

Multiple mechanisms may contribute to the BMP-7-mediated enhancement of VEGF-A expression observed in the present study. BMP-7 primarily exerts its biological effects via the BMP/Smad signaling pathway. The binding of BMP to the type II BMP receptor (BMPR-II) activates anaplastic lymphoma kinase (ALK 2, ALK 3, or ALK 6), leading to the phosphorylation of SMAD 1, SMAD 5, and SMAD 8 [41,42] and transcription of proliferation-related genes such as cyclin D1 and c-Myc as well as anti-apoptotic genes, such as Bcl-2, in MSCs [73]. BMP-7 may also stimulate VEGF secretion and activate survival pathways including AKT and ERK, thereby enhancing MSC viability and angiogenic activity [74,75]. BMP-7 has also been reported to enhance VEGF-mediated signaling by increasing cellular responsiveness to VEGF and upregulating VEGF receptor expression, particularly VEGFR2 [42]. In addition to its direct signaling effects, BMP-7 indirectly modifies MSC behavior by promoting the secretion of angiogenic cytokines, including basic fibroblast growth factor (bFGF) and angiopoietin-1 [76]. These synergistic actions of BMP-7 promote capillary sprouting, vascular stabilization, and ECM remodeling. In addition, WJMSCs exhibit intrinsic pro-angiogenic properties through their secretome and ECM components. A recent study demonstrated that extracellular matrix derived from WJMSCs promotes angiogenesis by activating the integrin αVβ3/c-Myc/P300 signaling axis, thereby enhancing VEGF expression and endothelial cell activity [77]. Collectively, these findings provide a plausible mechanistic explanation for the enhanced VEGF-A expression observed following BMP-7 treatment in the present study. The observed increase in VEGF-A expression supports the hypothesis that BMP-7 modulates the paracrine activity of WJMSCs within a tissue-specific extracellular matrix, thereby generating a microenvironment that is more conducive to vascular regeneration than the scaffold alone.

The present study extends our previous findings that BMP-7 enhances the dentinogenic differentiation of WJMSCs cultured within DHDP [40] by demonstrating increased WJMSC viability and increased VEGF-A-associated pro-angiogenic expression within the same tissue-engineered construct. Similarly, Liang et al. demonstrated that BMP-7 promoted stem cell recruitment, odontogenic differentiation, and angiogenesis in an endogenous pulp regeneration model, resulting in the formation of vascularized pulp-like tissue in vivo [43]. Collectively, these findings suggest that BMP-7 may simultaneously regulate multiple regenerative pathways required for functional pulp regeneration, including cellular survival, odontogenic differentiation, and angiogenic signaling, rather than promoting only a single biological outcome. Such coordinated regulation is biologically relevant because regeneration of the pulp–dentin complex requires concurrent vascular, neural, and odontogenic development to restore a functional pulp–dentin complex. The present DHDP+WJMSC+BMP-7 construct represents an important step towards the development of a clinically translatable tissue-engineered pulp substitute, particularly for mature permanent teeth where current cell-homing regenerative procedures remain less predictable.

Despite the findings, several limitations should be acknowledged. First, functional angiogenesis was not directly evaluated, as increased *VEGFA* gene and VEGF-A protein expression indicates angiogenic potential but does not demonstrate angiogenic function or vascular formation. Furthermore, the assessment of angiogenic potential relied primarily on a single angiogenic marker, VEGF-A. Although VEGF-A is a key regulator of angiogenesis, reliance on a single marker limits comprehensive evaluation of this complex multistep process. Future studies should therefore incorporate additional markers associated with endothelial activation, angiogenic signaling, and vascular maturation, such as CD31, FGF2, ANGPT1, VEGFR2, and PDGF, to provide a broader assessment of angiogenic potential [46]. Moreover, IHC primarily provides qualitative protein detection; therefore, quantitative methods such as ELISA [78], flow cytometry [79], or tube formation assay [43] should be incorporated into future investigations.

Second, the present study was conducted in an in vitro environment, which may not fully replicate the complex biological conditions of the pulp chamber in vivo, including vascular supply, immune responses, and host–tissue interactions. Hence, in vivo implantation studies should be conducted to evaluate further the angiogenic potential of the experimental tissue-engineering construct employed in this study. However, regeneration of a functional pulp–dentin complex extends beyond vascularization. Although the present study focused on angiogenic potential, odontogenic and neurogenic differentiation were not evaluated. Future studies should therefore assess odontogenic differentiation and mineralization, as well as neurogenic outcomes, alongside angiogenic responses to determine whether the construct can support coordinated pulp–dentin complex regeneration. Long-term success will also depend on appropriate odontoblast-like cell organization along the dentinal wall, immune homeostasis, and stable neurovascular integration following implantation. Further investigations should therefore evaluate long-term pulp tissue function and immune regulation using appropriate endothelial, odontogenic, and neural differentiation or co-culture models, followed by orthotopic in vivo studies.

Third, decellularization was confirmed qualitatively by H&E, DAPI, and FESEM. Future studies should incorporate residual DNA quantification, biochemical characterization of retained ECM components including collagen, and mechanical testing to provide a more comprehensive evaluation of decellularization quality and scaffold functionality. Moreover, scaffold architecture was evaluated primarily by pore-size measurements, without complementary porosity analysis, thereby limiting assessment of pore interconnectivity and its influence on cellular infiltration. Pore size was expressed as area rather than diameter because of the irregular morphology of the scaffold pores; however, this limits direct comparison with previous studies reporting pore diameter and should therefore be interpreted cautiously [80]. Although no standardized method for pore-size evaluation currently exists [81], future studies should incorporate quantitative porosity analysis and three-dimensional characterization techniques such as micro-computed tomography (micro-CT) to provide a more comprehensive evaluation of scaffold microarchitecture. The predominantly peripheral distribution of WJMSCs with limited infiltration into the scaffold core may reflect the combined influence of scaffold degradation, swelling behavior, pore architecture, pore size, and porosity [57], which can impair cellular migration and restrict nutrient and oxygen transport within deeper scaffold regions [82]. Strategies to improve cell-seeding efficiency and distribution should also be incorporated. Dynamic cell-seeding approaches such as rotational seeding [83], vacuum-assisted seeding [84], and hybrid rotational–vacuum approaches [85] may improve cellular infiltration and promote more homogeneous scaffold repopulation.

Finally, the biological materials were obtained from donors across different age ranges. As donor age was not investigated as an independent variable, potential age-related and inter-donor variability in the cellular and extracellular matrix characteristics of the tissues may have contributed to biological heterogeneity in the present findings. Future studies should therefore consider controlling or stratifying donor-related variables, including age, when evaluating scaffold and cellular outcomes.

Other than experimental limitations, several biological and translational challenges remain. In the present study, BMP-7 was administered directly in the culture medium as a controlled experimental approach and does not represent a definitive clinical delivery strategy. For translational applications, localized delivery systems, such as BMP-7-loaded gelatin hydrogel sheets [86], PLGA scaffolds [87], and chitosan microparticles [88], may facilitate controlled and sustained BMP-7 release within the regenerative construct. Release kinetics should be characterized to determine the concentration and duration of BMP-7 exposure within the scaffold [87], as the optimal dose for angiogenic signaling may differ from that required for odontogenic differentiation or long-term tissue maturation. Future studies should therefore evaluate both dose–response relationships and release profiles to optimize BMP-7 delivery for coordinated pulp–dentin regeneration. From a translational perspective, routine clinical application of tissue-engineered pulp constructs will require reliable sources of decellularized scaffolds, standardized manufacturing protocols, scalable stem cell production, and regulatory compliance to ensure safety, reproducibility, and cost-effectiveness. Advances in patient-specific scaffold fabrication or injectable extracellular matrix systems may further improve adaptation of engineered constructs to complex root canal anatomies and facilitate future clinical implementation. Additionally, the clinical delivery of growth factors such as BMP-7 may present logistical and economic challenges in routine dental practice.

From the clinician’s perspective, the ultimate objective of regenerative endodontics is not merely radiographic healing but regeneration of vital pulp tissue capable of maintaining dentin homeostasis, immune defense, sensory function, and physiological responses to injury. In contrast to current cell-homing regenerative procedures, which rely on endogenous stem-cell recruitment, a pre-engineered pulp construct could potentially be implanted into a disinfected root canal to provide a biologically active regenerative microenvironment. Such an approach may improve the predictability of regenerative treatment in mature permanent teeth, where endogenous regenerative capacity is more limited [11]. However, substantial preclinical validation and well-designed clinical trials will be required before routine clinical application can be considered.

Consequently, the next step towards clinical translation will require well-designed in vivo studies, followed by early-phase clinical trials, to evaluate vascular integration, functional pulp regeneration, and the long-term safety of the DHDP+WJMSC+BMP-7 construct.

## 4. Materials and Methods

### 4.1. Sample Collection

This study was approved by the Universiti Kebangsaan Malaysia (UKM) Human Research Ethics Committee (UKM PPI/111/8/JEP-2023-318). All protocols used in this study complied with the Declaration of Helsinki. An overview of the study design and proposed translational application was illustrated in Figure 10.

Sample size calculation was performed based on the difference in means and the standard error of the mean (SEM) of *VEGFA* relative mRNA expression, which were 0.13 and 0.1, respectively, as reported in a previous study [89]. PS software version 3.1.6 [90] was used to calculate the sample size based on a comparison of two means (independent t-test). Six samples were required in each group (untreated and experimental groups) (*n* = 6) to detect a difference of 0.1 with 80% power at an alpha level of 0.05.

Human umbilical cords were collected postnatally with written informed consent from the mothers. The inclusion criteria were as follows: full-term healthy infants; mothers aged between 18 and 35 years; and negative maternal screening for infectious diseases (e.g., HIV, hepatitis B, sexually transmitted infections) and non-communicable conditions (e.g., hypertension, gestational diabetes mellitus). A 10 cm length of umbilical cord was collected and processed within 24 h of collection.

Human dental pulp tissues were collected from non-cavitated teeth, extracted for orthodontic purposes from patients aged 11 to 30 years. Written consent was obtained from all participants or their legal guardians before tooth collection. Pulp tissues were aseptically extirpated, preferably immediately or within 24 h of extraction, and stored in 1× phosphate-buffered saline (PBS) (Solarbio, Beijing, China) at –20 °C (Figure 11).

### 4.2. Isolation and Characterization of WJMSCs

Wharton’s jelly was enzymatically digested with 0.3% Type I Collagenase at 37 °C to isolate WJMSCs. Cells were cultured in Dulbecco’s Modified Eagle Medium/Nutrient Mixture F-12 Ham (DMEM/F12) (Gibco, Thermo Fisher Scientific, Waltham, MA, USA) supplemented with 10% fetal bovine serum (FBS) (Capricorn Scientific, Ebsdorfergrund, Germany), 1% antibiotic-antimycotic (AA) (Gibco, Thermo Fisher Scientific, MA, USA), and 1% L-ascorbic acid (Sigma Aldrich, St. Louis, MO, USA). The media was changed every two to three days. WJMSCs were passaged at 80% confluency. Cells at passage 4 were trypsinized, counted, and subsequently characterized. The WJMSCs at passage 4 were visualized under an inverted phase-contrast light microscope at 40× magnification. Mesenchymal stem cell surface markers (CD73^+^, CD90^+^, CD105^+^; CD34^−^, CD45^−^, HLA-DR^−^) and stemness markers (NANOG, OCT4, SOX2) (Table 2) were analyzed using quantitative reverse transcriptase polymerase chain reaction (qRT-PCR). Trilineage differentiation assays (adipogenic, osteogenic, and chondrogenic) were performed to confirm their multipotency.

### 4.3. Preparation and Characterization of DHDP

Pulp tissues were decellularized using chemical treatment with 1% Triton X-100 (Vivantis, Subang Jaya, Malaysia) and 1% sodium dodecyl sulfate (SDS) (Bendosen, Damansara, Malaysia) in an incubator shaker at 37 °C. This protocol was adapted from Song et al. [44] and modified from Traphagen et al. [91] and Datta et al. [92], as outlined in Table 3. Decellularized tissues were disinfected in 1× PBS containing 1% AA for 24 h [93], then washed three times in 1× PBS and stored at –20 °C until use. Complete decellularization was confirmed via hematoxylin and eosin (H&E) (Leica, Wetzlar, Germany) and 4′,6-diamidino-2-phenylindole (DAPI) (BioLegend, San Diego, CA, USA) staining.

Ultrastructural features of the DHDP were examined using field emission scanning electron microscopy (FESEM) (Carl Zeiss, Thuringia, Germany) at an accelerating voltage of 3 kV. Samples of human dental pulp and DHDPs were fixed in 2.5% glutaraldehyde solution for 24 h, dehydrated sequentially in ethanol, and freeze-dried. The dried samples were then mounted on a metal stub and coated with a layer of iridium using a sputter coater under an argon atmosphere prior to microscopic analysis. Finally, the samples were vacuum-sealed, and their morphology was examined using a FESEM at an accelerating voltage of 3 kV.

Pore size measurements were derived from FESEM images using the area measurement tool in ImageJ software (Version 1.54r), by utilizing the “adjust threshold tool” to auto-highlight the pores with border refinement and calculate the total area of pores. The analysis was repeated twice for each image to obtain the average total pore area for the samples. Pore area was selected because the pores observed in the FESEM images were irregular and frequently non-circular. Reporting an equivalent diameter would require an assumption of circular geometry that may not adequately represent the morphology of the native DHDP scaffold. To ensure reliability and consistency in pore size measurements, inter- and intra-examiner calibration was conducted. A set of randomly selected SEM images was used for calibration. Two examiners (P.W.Y. and N.I.M.A.) independently measured the pore area of the selected images using the same ImageJ protocol as described above. For intra-examiner calibration, the primary examiner (P.W.Y.) repeated the measurements on the same set of images after a 4-week interval to minimize recall bias. The measurements obtained from the two examiners and the repeated measurements were then compared, and agreement was assessed using Cohen’s kappa coefficient.

Mechanical properties, such as water absorption and degradation rate of DHDP, were assessed. The DHDP samples were oven-dried for 30 min at 45 °C in an incubator oven. The samples were then weighed and recorded as W_D_. Subsequently, the samples were immersed in 1 mL of 1× PBS (pH 7.22) in each well of a 24-well plate for 24 h at room temperature. After removing the samples from PBS, excess liquid was blotted off with filter paper, and the samples were weighed immediately as WW. Each measurement was performed twice. The swelling ratio was calculated using the following formula, where W_W_ and W_D_ represent the weights of the wet and dry scaffolds, respectively [51]:

$$Swelling\;ratio\left(\%\right)=\frac{W_{W}-W_{D}}{W_{D}}\times 100$$

To determine the degradation rate, excess liquid from DHDP samples was removed using filter paper. The sample was weighed and recorded as W_D_. The samples were then immersed in 1× PBS (pH 7.22), using the minimum volume-to-scaffold ratio (30 µL: 1 mg) as proposed by ISO 13781:2017 [94], in each well of a 24-well plate sealed with film and incubated at 37 °C. PBS was changed every two to three days. On days 7, 14, and 21, the samples were removed from the wells, excess liquid was blotted off with filter paper, and the samples were weighed immediately as W_W_. Each measurement was repeated twice. The degradation rate was calculated using the formula where W_W_ and W_D_ are the weights of the wet and dry scaffolds, respectively [51]:

$$Degradation\;rate\left(\%\right)=\frac{W_{D}-W_{W}}{W_{D}}\times 100$$

### 4.4. Optimization of BMP-7 Concentration

The effective concentration of BMP-7 was determined using two-fold (6.25, 12.5, 25, 50 ng/mL) and ten-fold (60, 70, 80, 90, 100 ng/mL) serial dilutions. WJMSCs (5 × 10^3^ cells) were seeded in a 96-well plate. The cells were cultured for 1 day, then treated with different concentrations of BMP-7 for 72 h in accordance with ISO 10993-5:2009, Part 5, including negative control wells treated with DMEM/F-12 + 10% FBS without BMP-7. WJMSC viability at each concentration was assessed using the Thiazolyl Blue Tetrazolium Bromide (MTT) assay (Sigma-Aldrich, St. Louis, MO, USA) by measuring the absorbance value at 570 nm with a microplate reader.

### 4.5. Cytotoxicity Testing of DHDP and BMP-7

DHDP leachate was prepared by incubating scaffolds in DMEM supplemented with 10% FBS at 37 °C for 72 h at an extraction ratio of 0.1 g/mL, in accordance with ISO 10993-12:2021 [95]. The cytotoxicity of BMP-7, DHDP, and DHDP + BMP-7 was assessed on 5 × 10^3^ WJMSCs in a 96-well plate using the MTT assay after 72 h of treatment, in accordance with ISO 10993-5:2009 [96] standards, by measuring the absorbance at 570 nm with a microplate reader, and the cell viability was calculated with the formula:

$$Cell\;viability\left(\%\right)=\frac{Absorbance\;of\;treated\;cells-Absorbance\;of\;blank}{Absorbance\;of\;control\;well-Absorbance\;of\;blank}\times 100\%$$

### 4.6. Viability of WJMSCs in DHDP

Disinfected DHDP samples were seeded with passage 5 WJMSCs (5 × 10^4^ cells in 20 µL DMEM/F-12 + 10% FBS per scaffold) and cultured for 21 days. Culture media were replenished every 2–3 days. The experimental group was repopulated within DHDP treated with 50 ng/mL BMP-7 in DMEM/F-12 + 10% FBS (DHDP+WJMSC+BMP-7), while the control group was repopulated within DHDP cultured with DMEM/F-12 + 10% FBS without BMP-7 (DHDP+WJMSC). Repopulation and cellular attachment were assessed using H&E, DAPI staining, and FESEM.

Cell viability of DHDP+WJMSC+BMP-7 and DHDP+WJMSC was evaluated using the Resazurin assay (Canvax Biotech, Valladolid, Spain) on days 7, 14, and 21, by reading the relative fluorescence unit (RFU) absorbance data at wavelengths of 570 nm and 600 nm, using a microplate reader. The percentage reduction of resazurin reagent was calculated from absorbance readings following the manufacturer’s instructions. The cell proliferation percentage was calculated following the formula:

$$\%\;Reduction\;of\;resazurin\;reagent=\frac{\left(E_{oxi}600\;\times \;A_{570})-(E_{oxi}570\;\times \;A_{600}\right)}{\left(E_{red}570\;\times \;C_{600})-(E_{red}600\;\times \;C_{570}\right)}\times 100$$

### 4.7. Angiogenic Gene Expression Analysis

After 21 days of WJMSC repopulation, total ribonucleic acid (RNA) from DHDP+WJMSC+BMP-7 and DHDP+WJMSC was extracted and processed using specific primer sequences (Table 2). The relative expression of each target gene mRNA was determined using the comparative delta-delta cycle threshold (ΔΔCt) method [97]:

$$\Delta \Delta Ct=\Delta Ct\left(VEGFA\right)-\Delta Ct\left(GAPDH\right)$$

$$Normalized\;data=2^{-\Delta \Delta Ct}$$

For qRT-PCR analysis, the human pre-mRNA sequence was obtained from the National Center for Biotechnology Information (NCBI) AceView program (https://www.ncbi.nlm.nih.gov/nuccore, accessed on 3 February 2025). All primers were designed to cross introns using the Primer3 website (https://primer3.ut.ee/, accessed on 3 February 2025) as shown in Table 4 [98].

### 4.8. Angiogenic Protein Expression Using Immunohistochemistry Analysis

On day 21, both DHDP+WJMSC+BMP-7 and DHDP+WJMSC were subjected to immunohistochemistry (IHC) staining using 5 µg/mL rabbit monoclonal anti-VEGF-A antibody (Abcam, Cambridge, UK) to evaluate the localization and expression of VEGF-A within the repopulated scaffold.

### 4.9. Statistical Analysis

Data were expressed as mean ± SEM and analyzed using SPSS software (Version 29.0). The selection of statistical tests was based on data distribution, the number of groups or time points being compared, and whether observations were independent or repeated. Parametric tests were applied when the relevant assumptions were satisfied, whereas non-parametric tests were used when these assumptions were not met. Comparisons between two independent groups were performed using an independent *t*-test or Mann–Whitney U test, as appropriate. Comparisons involving more than two groups or time points were performed using one-way ANOVA with Tukey’s post hoc test or the corresponding non-parametric tests (Kruskal–Wallis or Friedman test), as appropriate. The statistical tests applied to each analysis are summarized in Table 5. Statistical significance was defined as *p* < 0.05.

## 5. Conclusions

BMP-7 significantly enhanced WJMSC proliferation and *VEGFA* gene and protein expression within DHDP scaffolds, while maintaining biocompatibility at a concentration of 50 ng/mL. Although cell distribution remained predominantly peripheral, suggesting that further optimization of scaffold architecture is required to improve cellular infiltration, the DHDP scaffold provided a favorable tissue-specific microenvironment for WJMSC survival. Collectively, these findings support the potential of combining BMP-7, WJMSCs, and DHDP as a tissue-engineering construct for pulp regeneration. However, functional angiogenesis was not directly evaluated, and further optimization of the construct, together with in vivo evaluation of vascular integration and long-term functional outcomes, will be essential before clinical translation can be considered.

## Figures and Tables

**Figure 1:**
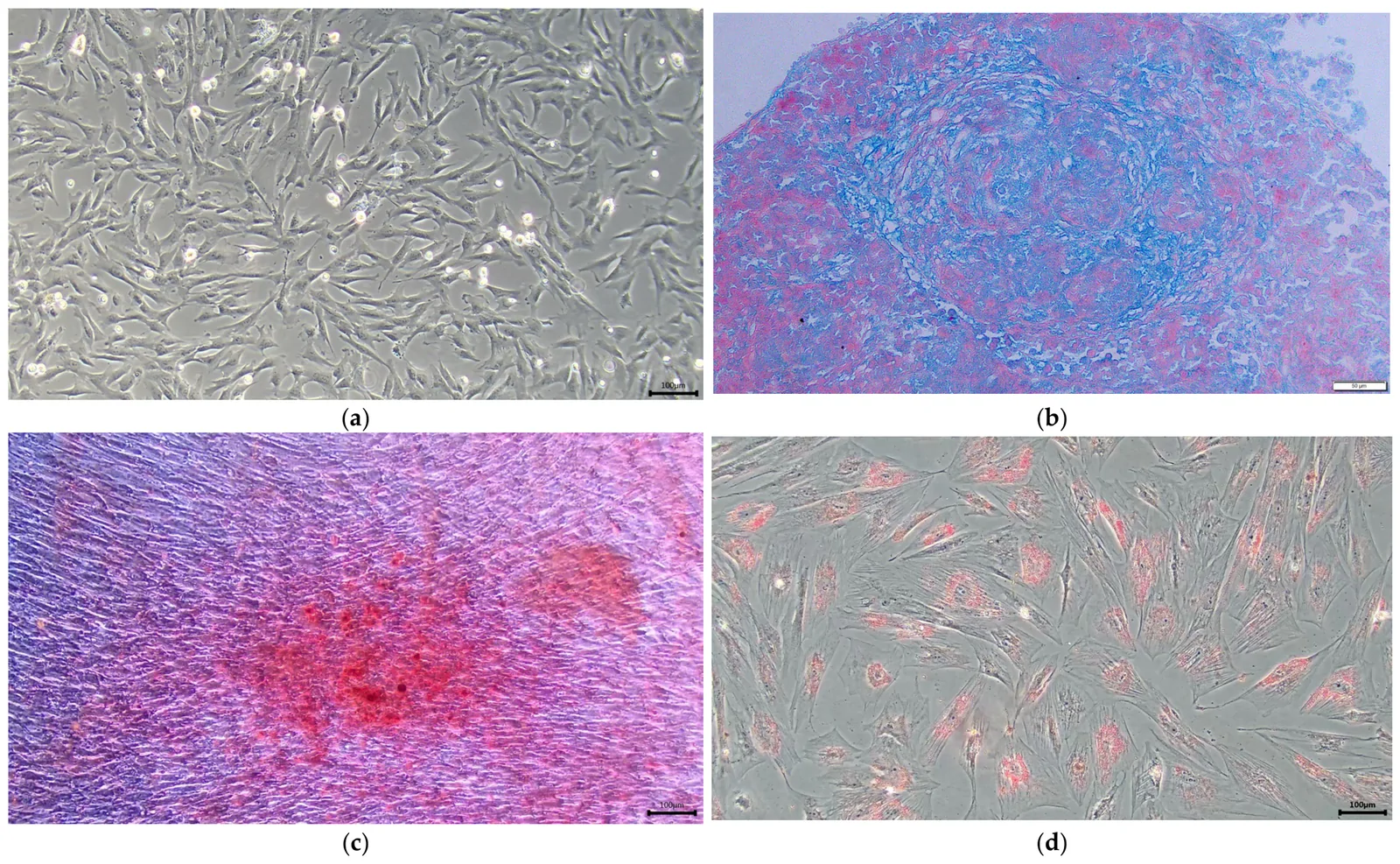
Characterization of WJMSCs. (**a**) Before induction, WJMSCs exhibited a uniform, spindle-shaped, fibroblast-like morphology and plastic adherence characteristic of MSCs (magnification, 40×). (**b**) Chondrogenic differentiation of WJMSCs was evidenced by glycosaminoglycan deposits stained blue and nuclei stained pink (magnification, 100×). (**c**) Osteogenic differentiation of WJMSCs was evidenced by positive, intense Alizarin Red S staining of mineralized nodules (magnification, 100×). (**d**) Adipogenic differentiation of WJMSCs was evidenced by positive Oil Red O staining of lipid droplets within the cytoplasm (magnification, 100×). Scale bars = 50 μm (**b**) and 100 μm (**a**,**c**,**d**).

**Figure 2:**
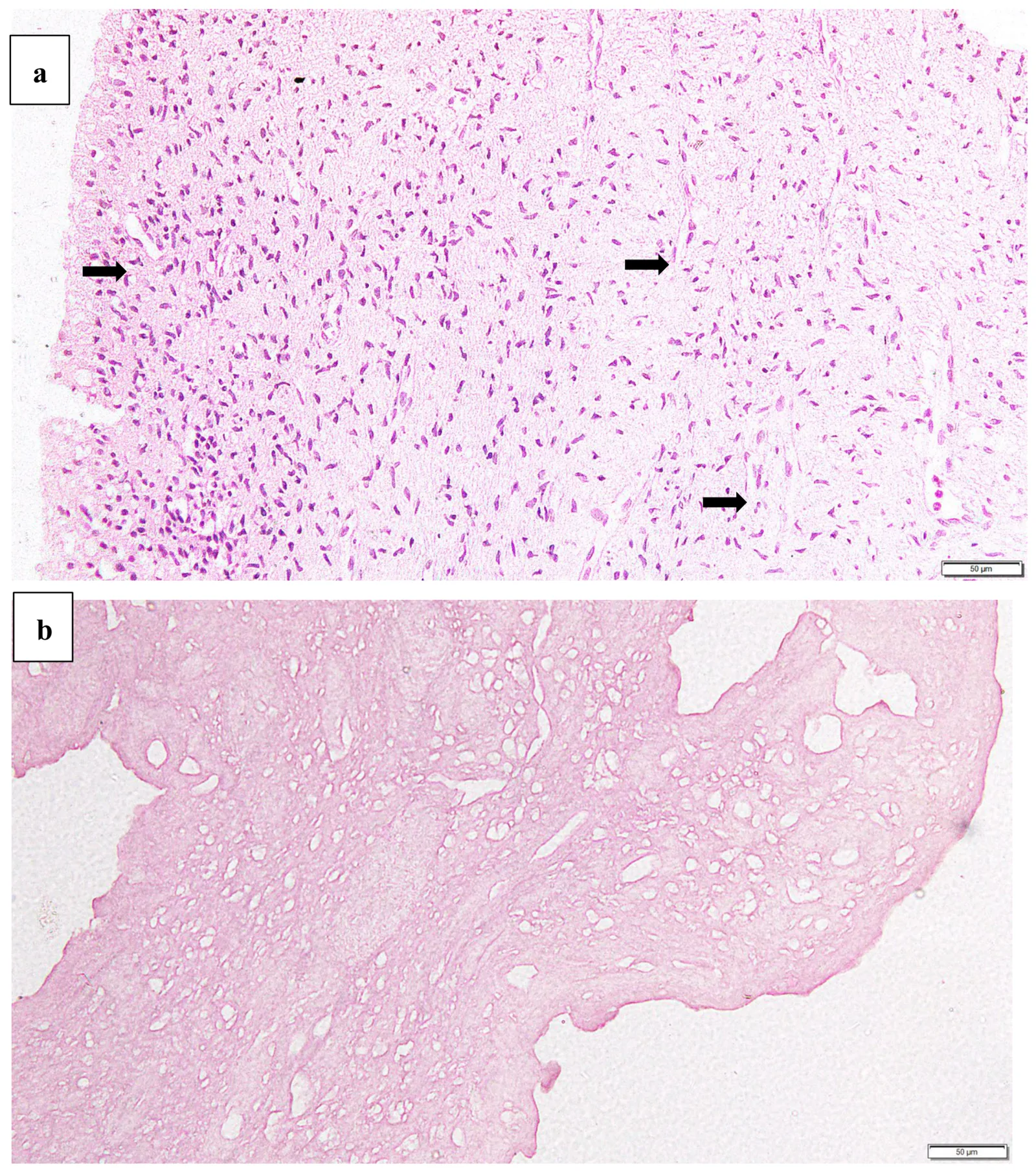
(**a**) H&E section of untreated pulp tissues with densely packed nuclei stained in dark purple, interspersed within the fibrous ECM. Black arrows point to endothelial cells lining the blood vessels. (200× magnification; scale bar = 50 μm). (**b**) The H&E section of DHDP demonstrated complete removal of cellular content from the preserved ECM, with a porous morphology (200× magnification; scale bar = 50 μm).

**Figure 3:**
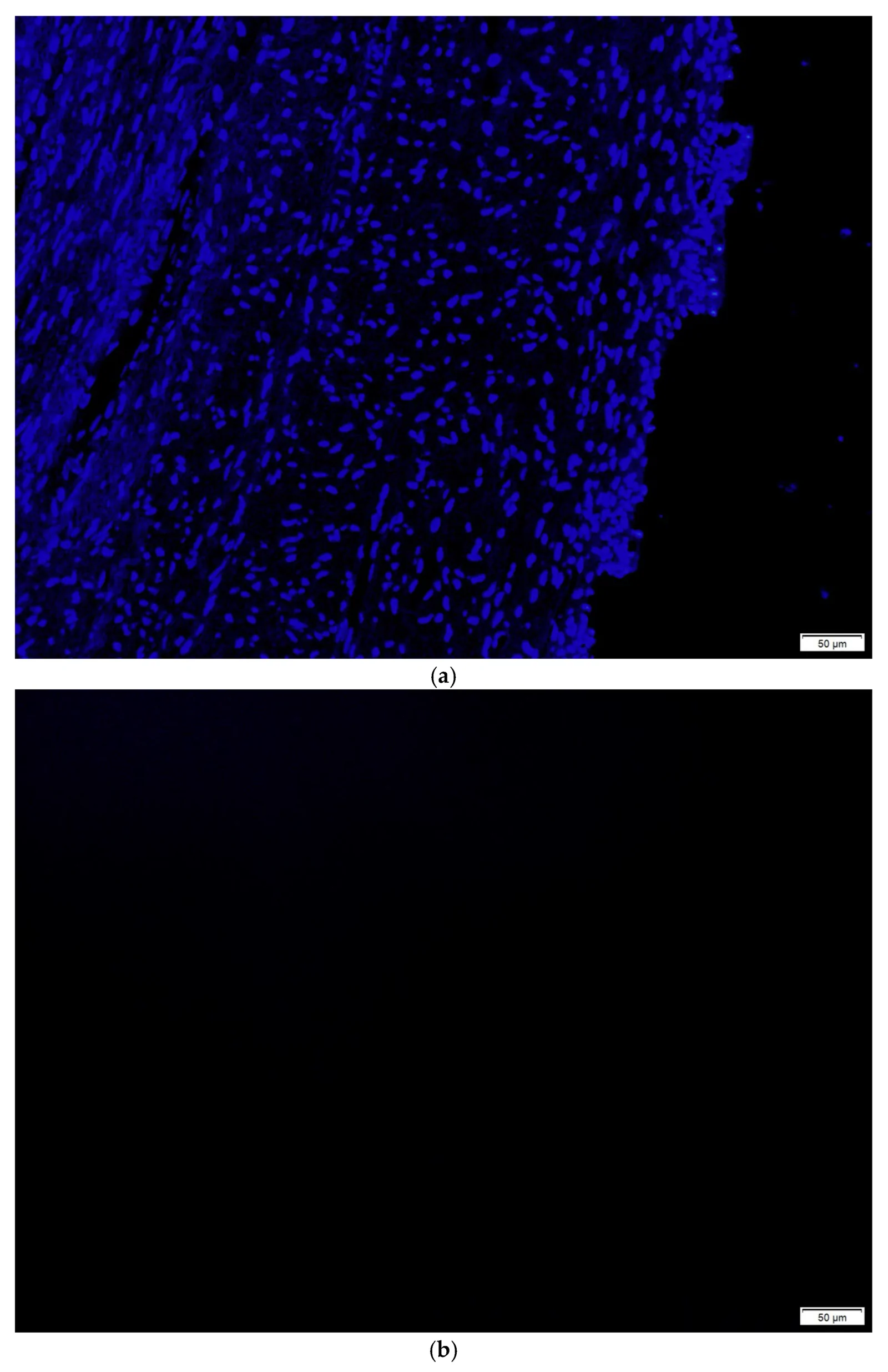
(**a**) DAPI staining of untreated pulp tissues shows dense and uniform distribution of nuclei, appearing as a fluorescent blue signal (200× magnification; scale bar = 50 μm). (**b**) DAPI staining of DHDP confirms successful removal of cellular components, indicated by a completely dark field with no nuclear fluorescence (200× magnification; scale bar = 50 μm).

**Figure 4:**
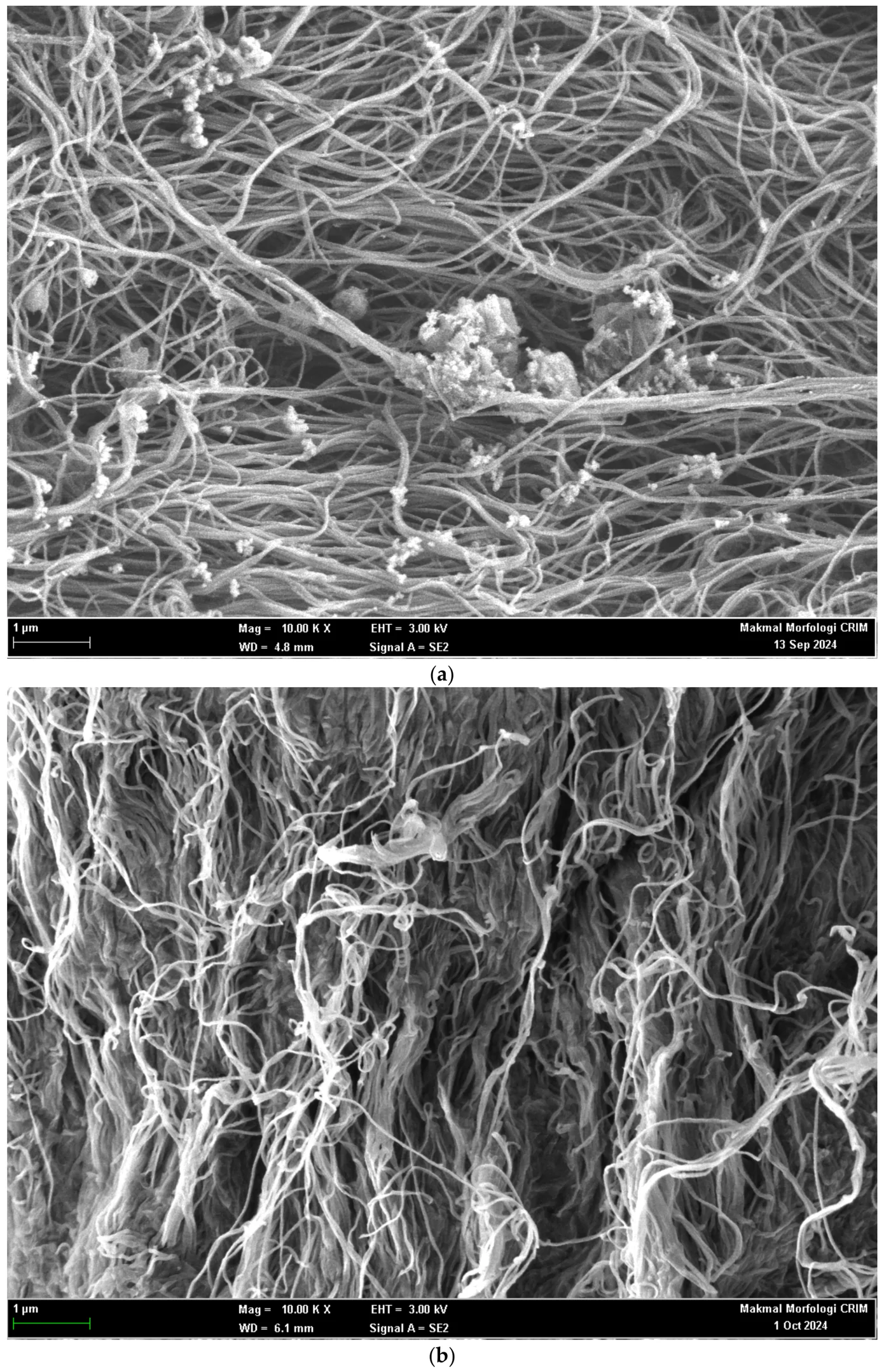
(**a**) FESEM imaging of untreated pulp tissues revealing densely interwoven fibrous ECM interspersed with numerous globular and particulate structures suggestive of cellular content (10,000× magnification; scale bar = 1 μm). (**b**) FESEM imaging of DHDP revealed a well-preserved yet more exposed, porous fibrous network lacking cellular content (10,000× magnification; scale bar = 1 μm).

**Figure 5:**
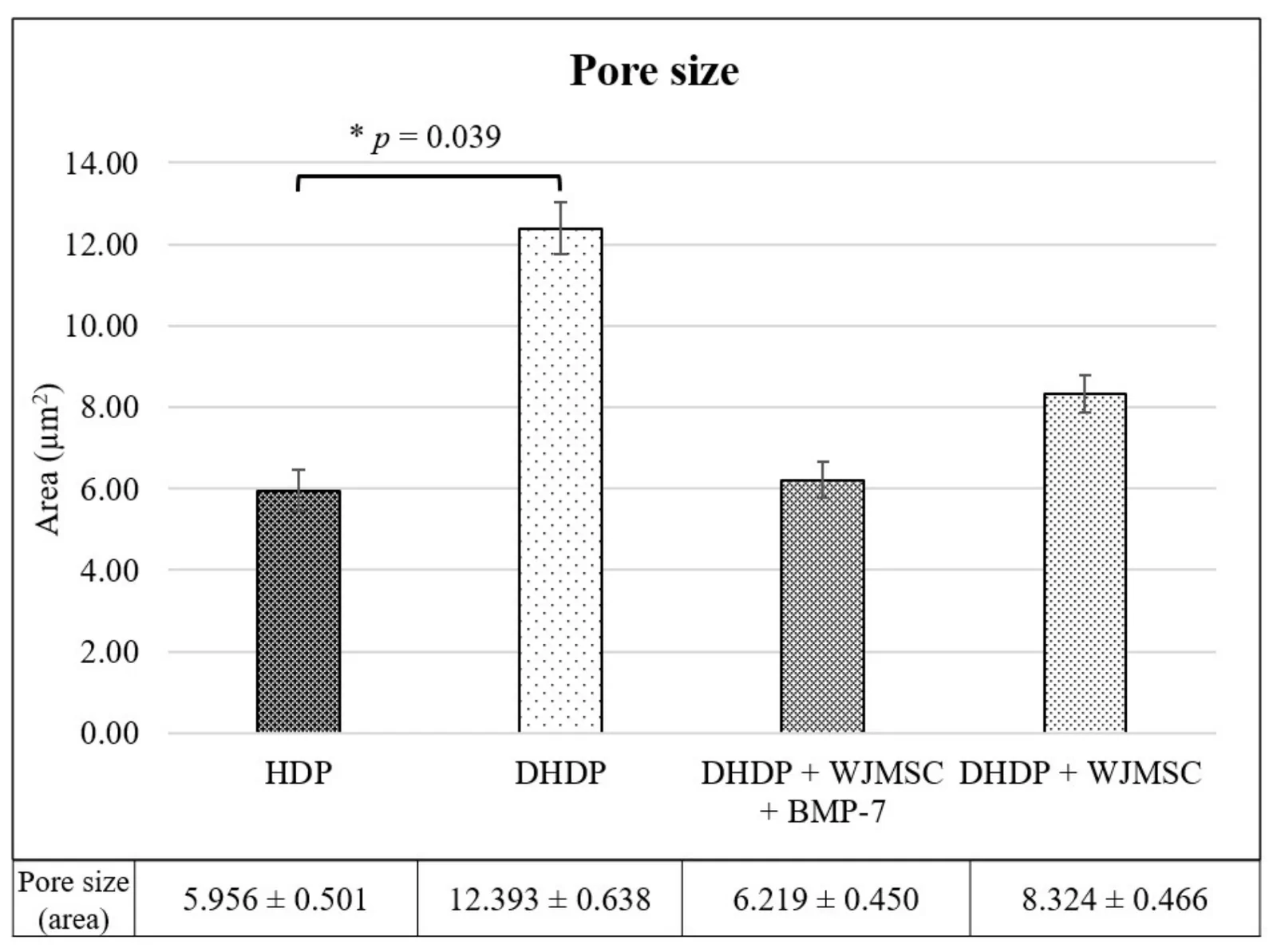
Pore size of samples (area, µm^2^). Pore size of DHDP was significantly larger when compared with untreated pulp tissues (HDP). Statistical difference (*) was observed between the untreated HDP and DHDP (*n* = 4, *p* < 0.05; Kruskal–Wallis test).

**Figure 6:**
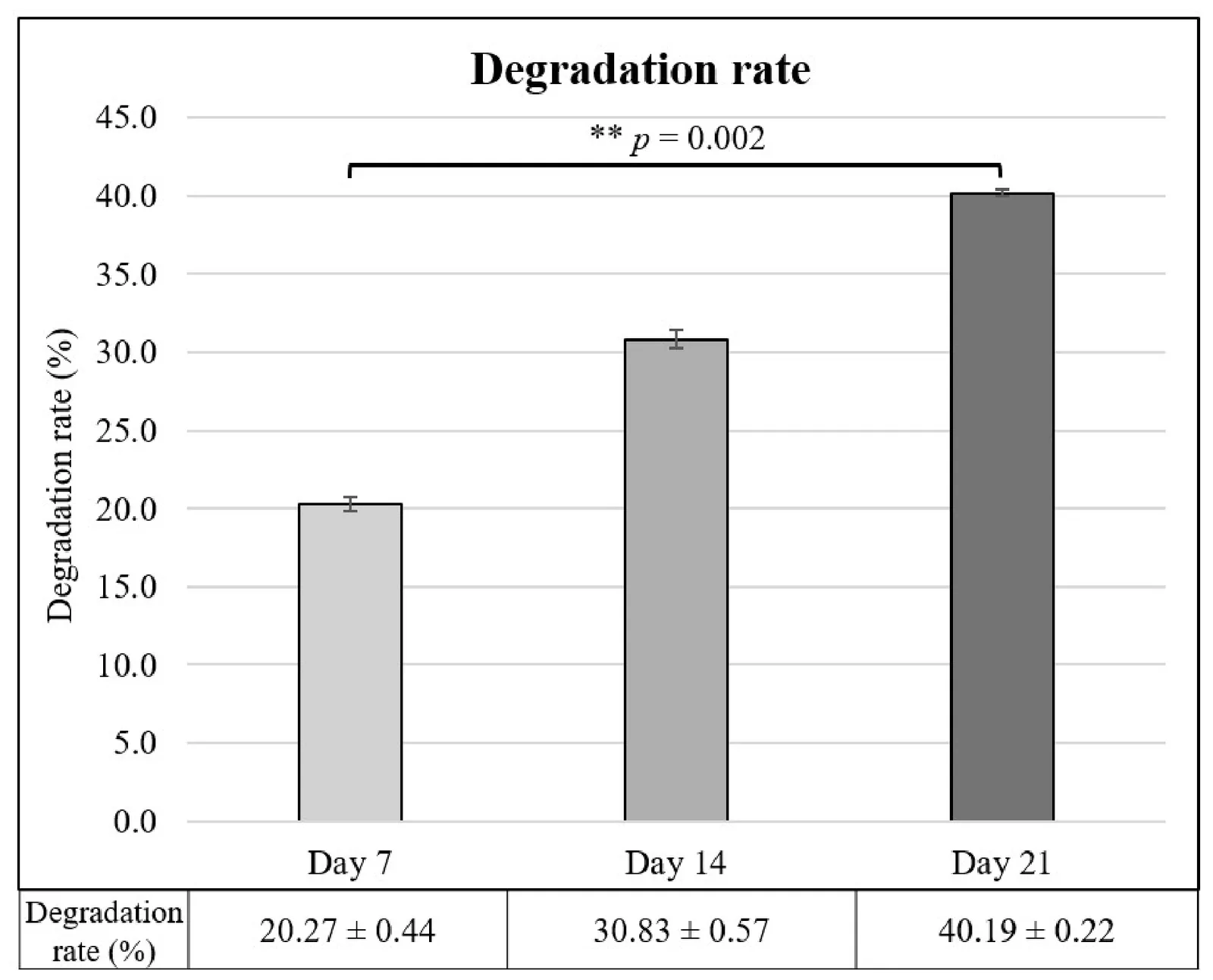
Degradation rate of DHDP (%). Statistical difference (**) was observed between the degradation rate on day 7 and day 21 (*n* = 6; *p* < 0.01; Friedman test).

**Figure 7:**
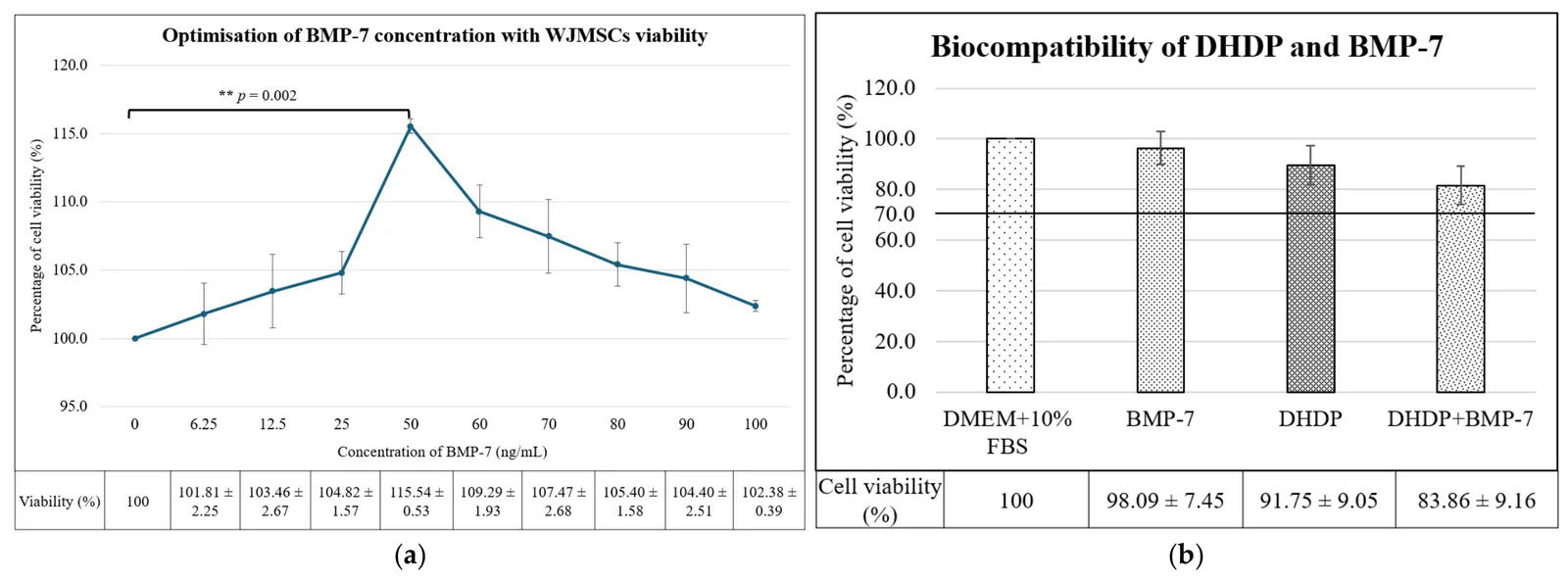

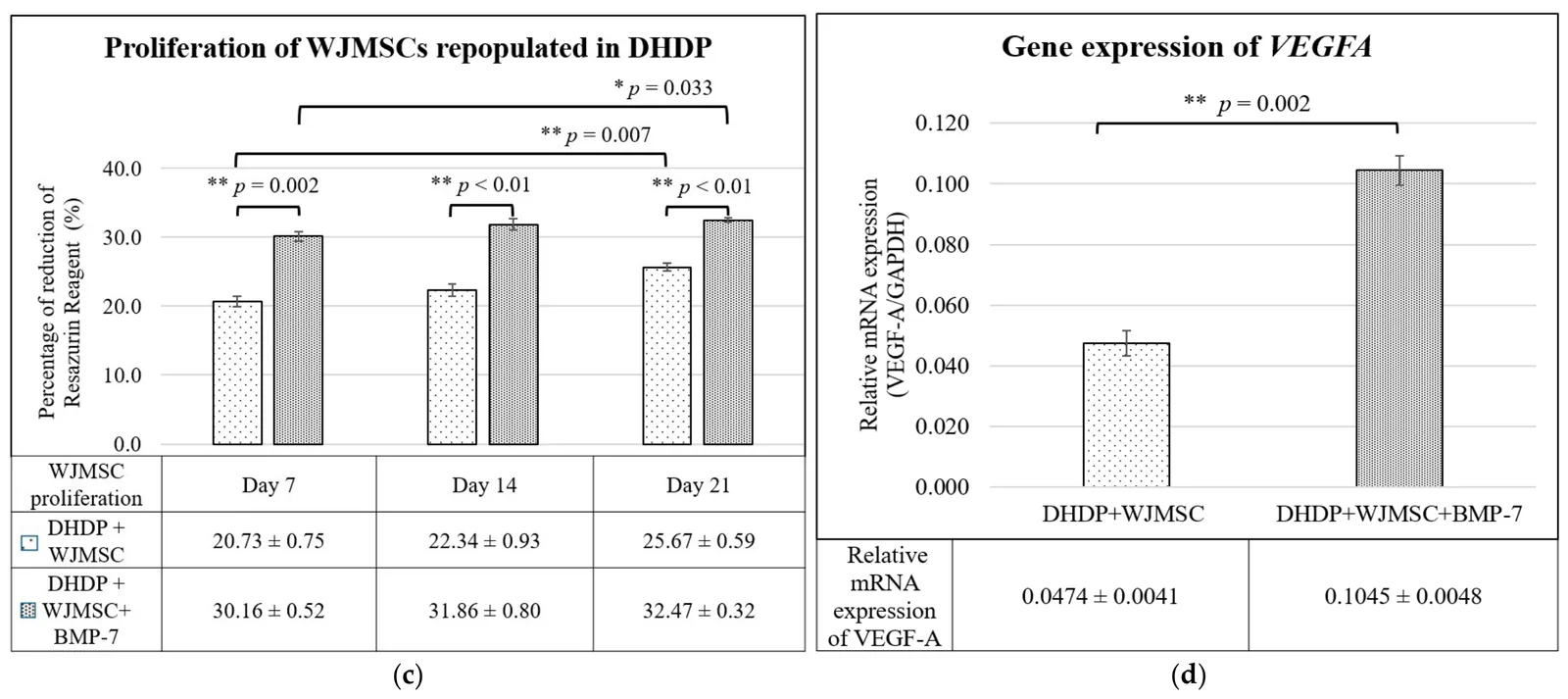
(**a**) Optimization of BMP-7 concentration based on WJMSC viability. The viability of WJMSCs treated with 50 ng/mL BMP-7 was significantly higher than that of the control (0 ng/mL), ** *p* < 0.01. A statistical difference (**) was observed between the viability of WJMSCs treated with 50 ng/mL BMP-7 and the control (*n* = 6; *p* = 0.002; Kruskal–Wallis test). (**b**) WJMSCs’ viability in different treatment groups was assessed for cytotoxicity using the MTT assay. Viability percentages above 70% indicate a non-cytotoxic effect of all treatment groups (*n* = 6; *p* > 0.05; Kruskal–Wallis test). (**c**) Comparing proliferation of WJMSCs in DHDP+WJMSC and DHDP+WJMSC+BMP-7. A statistical difference (**) was observed between the two groups at the same time point (*n* = 6; *p* < 0.01; Mann–Whitney U test for day 7, independent *t*-test for day 14 and day 21). A statistical difference (**) was observed within the DHDP+WJMSC group between day 7 and day 21 (*n* = 6; *p* < 0.01; Kruskal–Wallis test). A statistical difference (*) was observed within the DHDP+WJMSC+BMP-7 group between day 7 and day 21 (*n* = 6; *p* < 0.05; one-way ANOVA and Tukey post hoc test). (**d**) Angiogenic gene expression of repopulated WJMSCs. Statistical difference (**) was observed between DHDP+WJMSC+BMP-7 and the control untreated DHDP+WJMSC (*n* = 6; *p* < 0.01; Mann–Whitney U test).

**Figure 8:**
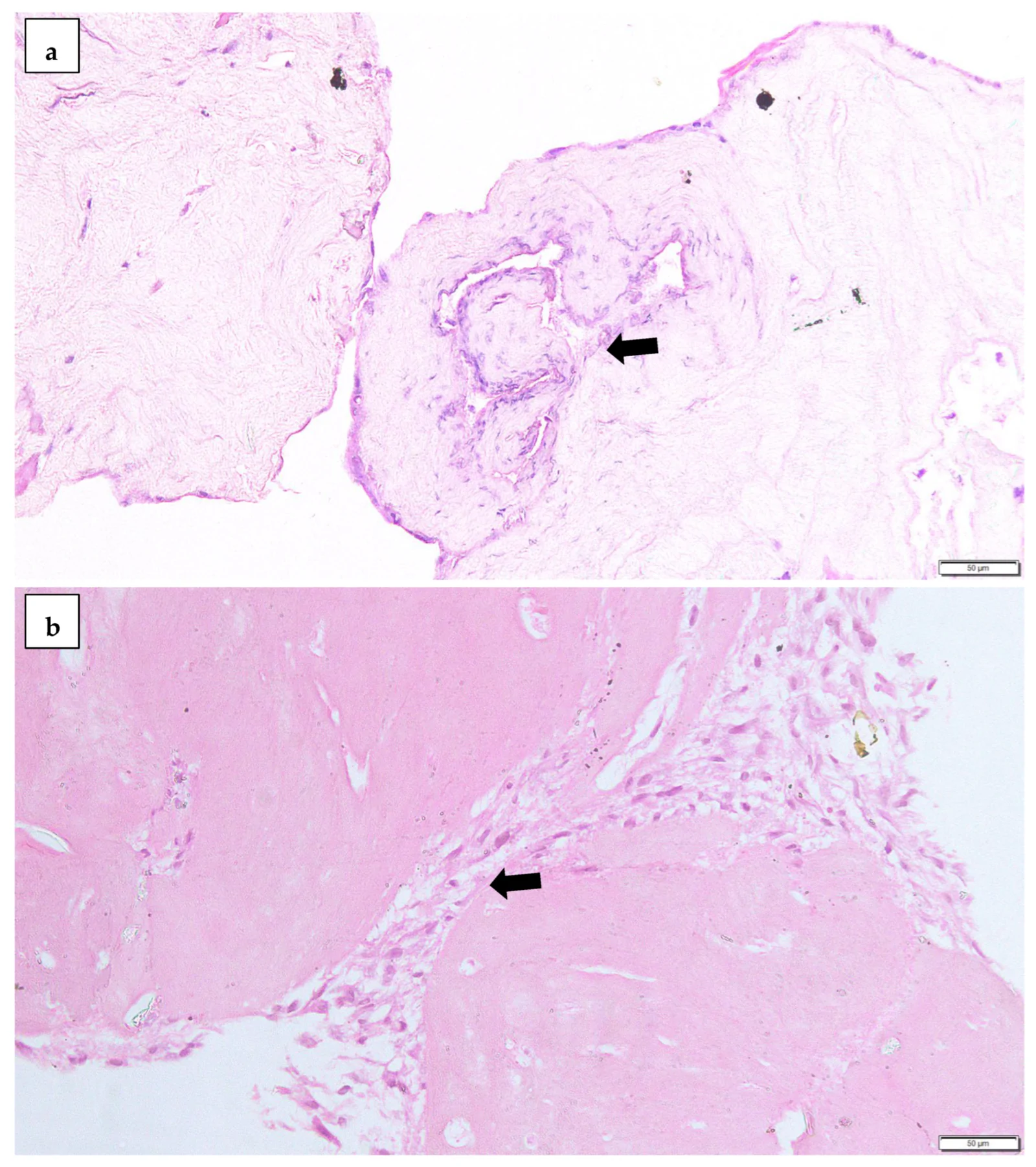
H&E section of (**a**) DHDP repopulated with WJMSCs (200× magnification; scale bar = 50 μm). (**b**) DHDP repopulated with WJMSCs and treated with BMP-7 (200× magnification; scale bar = 50 μm). Black arrows indicate representative repopulated cellular areas within the DHDP. Cellular repopulation was observed predominantly toward the scaffold periphery, with limited cellular infiltration into deeper scaffold regions in both groups.

**Figure 9:**
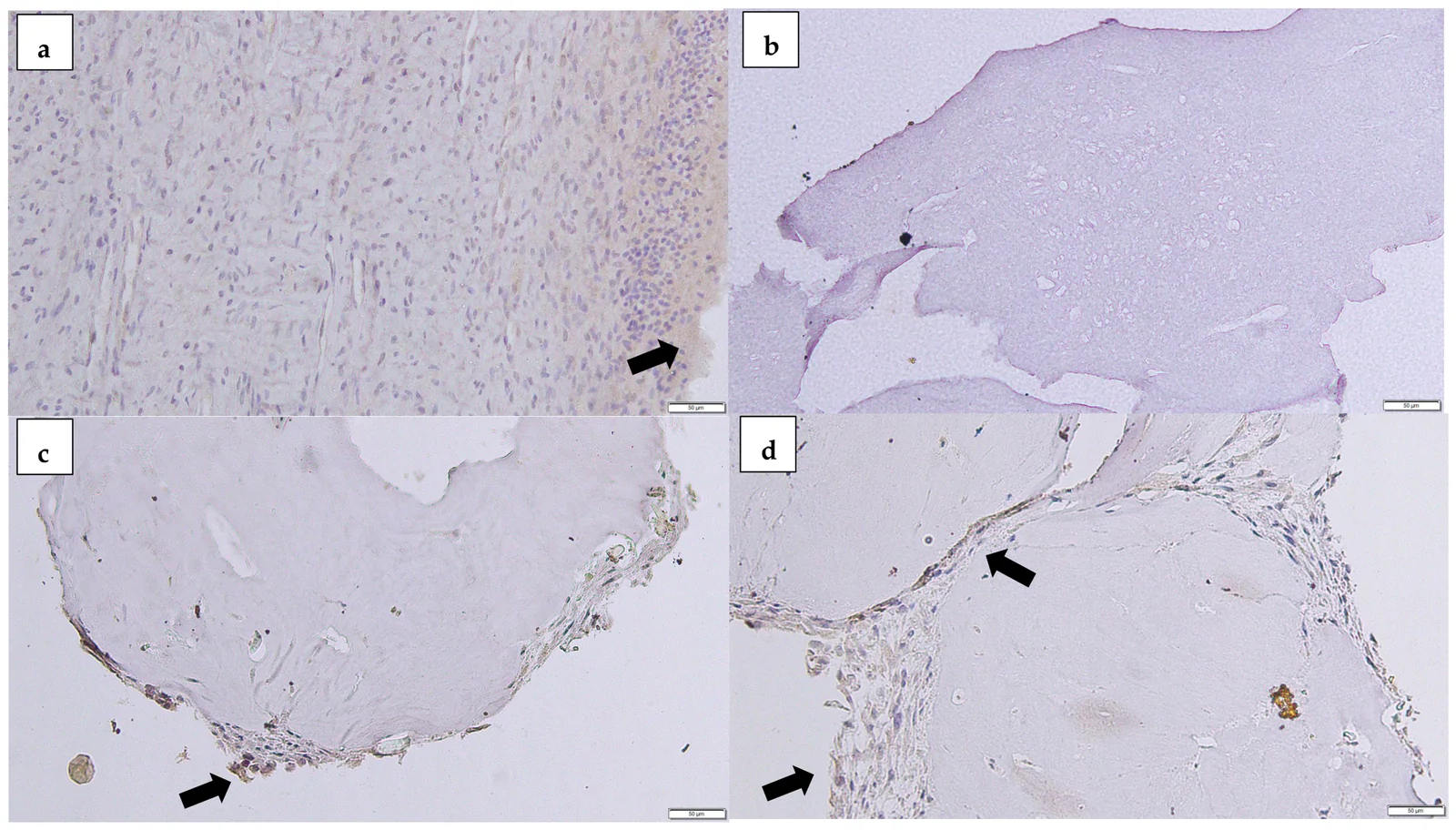
IHC staining of (**a**) Human dental pulp showing immunoreactivity, characterized by strong brown chromogenic staining (200× magnification; scale bar = 50 μm), (**b**) DHDP showing no detectable brown chromogenic staining, consistent with the absence of VEGF-A-expressing cells (200× magnification; scale bar = 50 μm), (**c**) DHDP+WJMSC with weak and diffuse brown staining indicating low levels of VEGF-A expression (200× magnification; scale bar = 50 μm), and (**d**) DHDP+WJMSC+BMP-7 with more intense and widespread brown cytoplasmic staining in cells distributed throughout the scaffold compared with DHDP+WJMSC (200× magnification; scale bar = 50 μm). Black arrows indicate representative areas of VEGF-A-positive brown chromogenic staining in (**a**,**c**,**d**).

**Figure 10:**
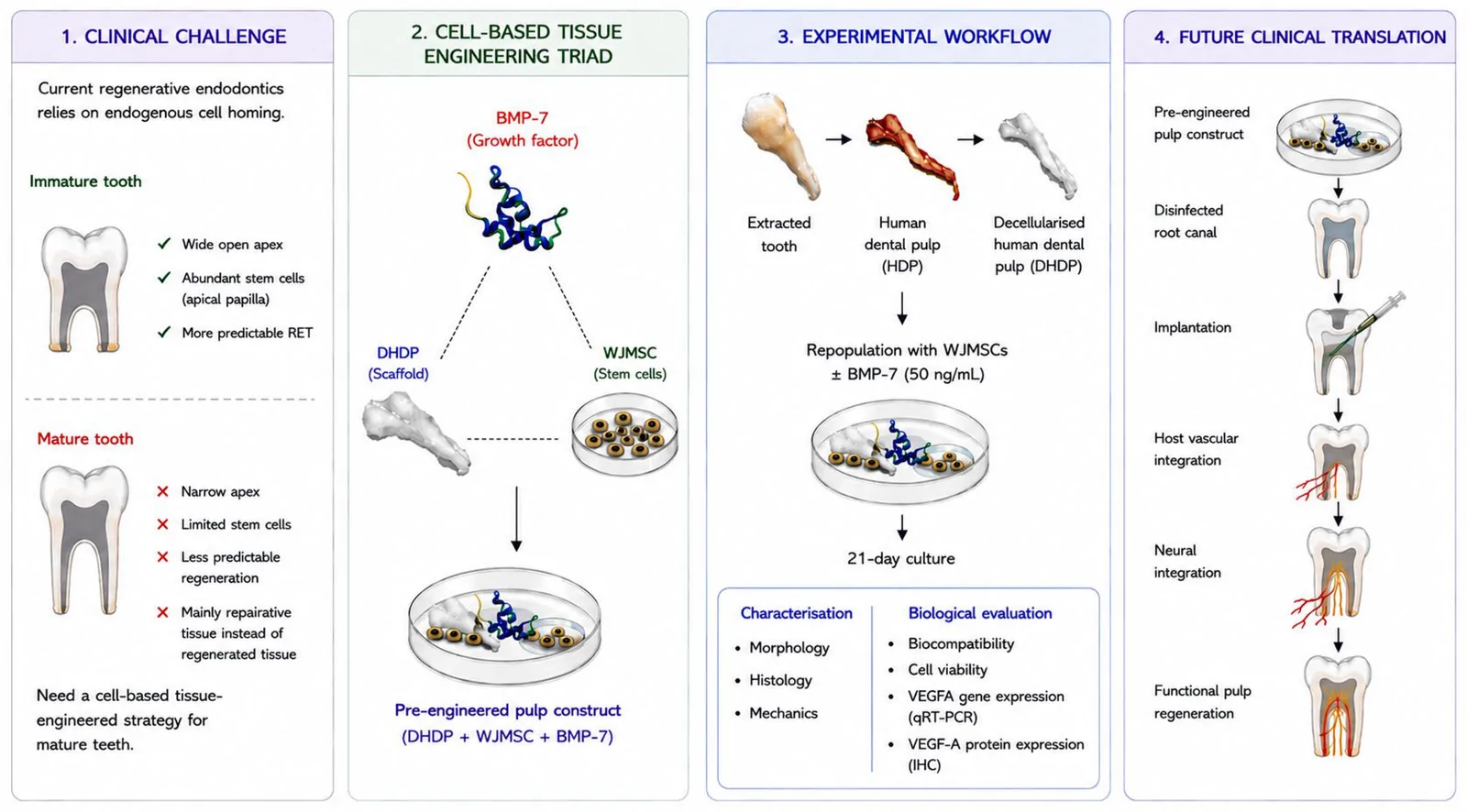
Overview of the study design and proposed translational application. A cell-based tissue-engineering triad consisting of WJMSCs, a DHDP scaffold, and BMP-7 was established to investigate the angiogenic potential of a tissue-engineered pulp construct. Human dental pulp obtained from extracted sound teeth was decellularized to obtain DHDP, then repopulated with WJMSCs and cultured in the presence or absence of 50 ng/mL BMP-7 for 21 days. Scaffold characterization included morphological, histological, and physical analyses, whereas biological evaluation comprised biocompatibility, cell viability, *VEGFA* gene expression, and VEGF-A protein expression. The final panel illustrates the proposed future clinical translation of the tissue-engineered construct for implantation into disinfected root canal systems to facilitate neurovascular integration and functional pulp regeneration.

**Figure 11:**
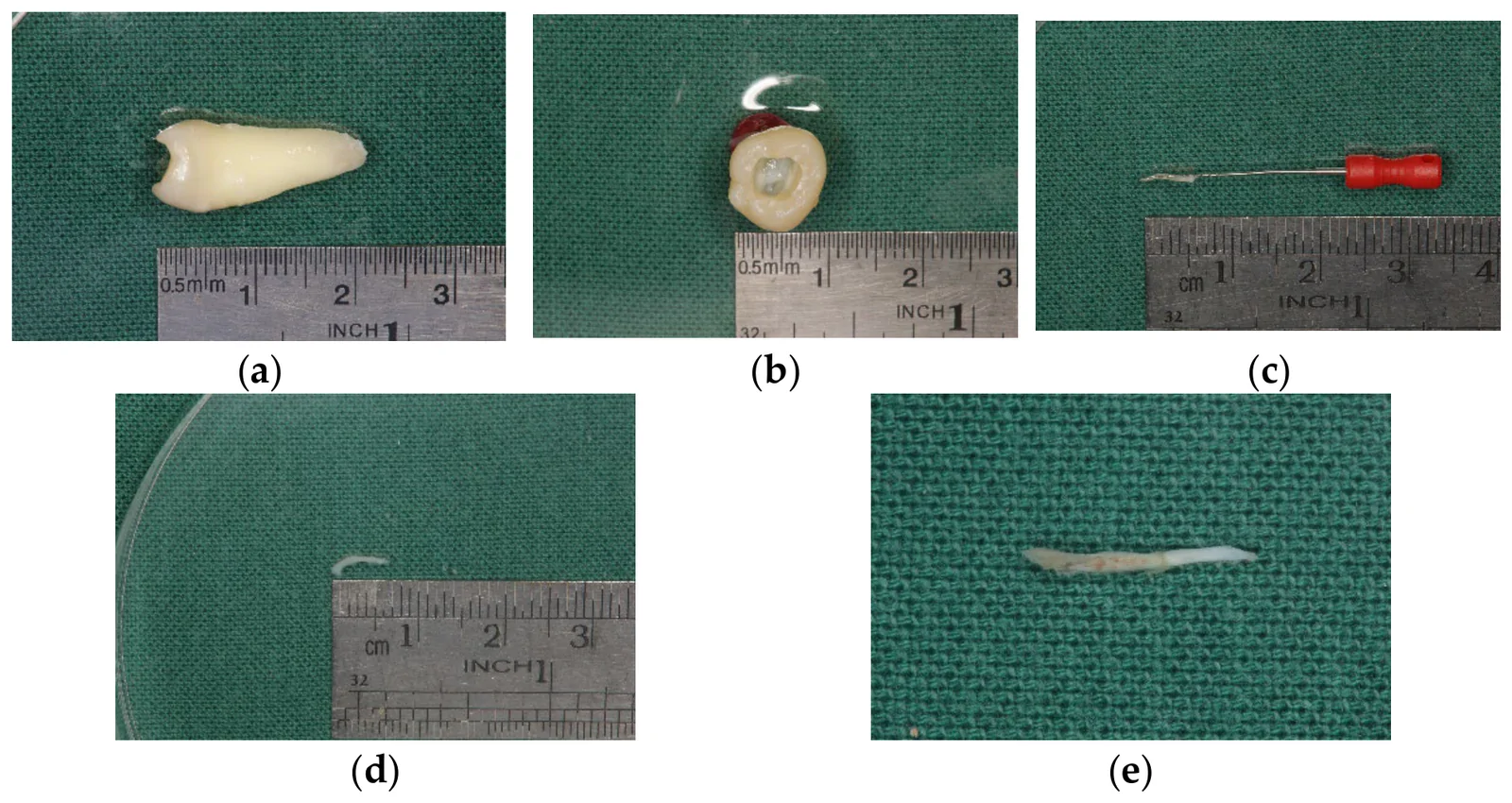
Extraction of pulp tissues. (**a**) Sound human premolar extracted. (**b**) Access cavity into the pulp chamber. (**c**) Extirpated pulp tissues on a sterile barbed broach. (**d**) The intact pulp tissues. (**e**) Pulp tissue appeared whitish to pinkish in color, indicative of vasculature in the pulp tissue.

**Table 1:** qRT-PCR relative gene expression of MSC surface markers and stemness markers.

Expression	Gene	Relative Gene Expression
Positive expression	NANOG	1.76 × 10^−5^ ± 4.71 × 10^−6^
	OCT4	7.58 × 10^−4^ ± 2.67 × 10^−5^
	SOX2	1.58 × 10^−5^ ± 5.90 × 10^−6^
	CD73	1.11 × 10^0^ ± 3.40 × 10^−1^
	CD90	1.93 × 10^−3^ ± 4.56 × 10^−4^
	CD105	4.34 × 10^−3^ ± 7.53 × 10^−4^
Very low expression	CD34	3.52 × 10^−5^ ± 1.33 × 10^−5^
	CD45	1.17 × 10^−5^ ± 6.88 × 10^−6^
	HLA-DR	2.03 × 10^−5^ ± 1.03 × 10^−5^

**Table 2:** List of gene and primer sequences involved in surface marker characterization using qRT-PCR.

Gene	Sequences	Product Size	Accession
NANOG	F: TCCTCCTGCCTGAGTCTCTC R: ATACAGGGCTAGGCTGGTGA	199	NM_024865
OCT4	F: GCAAAGCAGAAACCCTCGTG R: AACCACACTCGGACCACATC	172	NM_002701
SOX2	F: ATGGGTTCGGTGGTCAAGTC R: ACATGTGAAGTCTGCTGGGG	166	NM_003106.4
CD73	F: CCAGCAGTTGAAGGTCGGAT R: CTGTCACAAAGCCAGGTCCT	196	NM_002526
CD90	F: TGGTGAGAAGAGCTGCTGTG R: CACACAGTGCCGCTCATTTC	122	NM_006288
CD105	F: GGCAGCGAGGTAGTGAAGAG R: CGAAGGATGCCACAATGCTG	174	NM_000118
CD34	F: CTCAGCTCAATCGCCTCCAT R: CAAGCCACCTCCCTTCTCTG	191	NM_001025109
CD45	F: ATGATTGCTGCTCAGGGACC R: TCTCCCCAGTACTGAGCACA	140	NM_002838
HLA-DR	F: GTCAATGTCACGTGGCTTCG R: TCCACCCTGCAGTCGTAAAC	149	NM_019111.5

**Table 3:** Decellularization protocol of human dental pulp tissue.

Chemical Solution	Duration at 37 °C	Reference
0.01 M Tris-HCl, and 1 mM EDTA (pH 8.2)	48 h	Traphagen et al. [91]
1% SDS	24 h	Song et al. [44]
1% Triton X-100	24 h	
1% SDS	24 h	
1% Triton X-100	24 h	
1% SDS	24 h	
1% Triton X-100	24 h	
1× PBS + 1% AA	24 h	Datta et al. [92]
1× PBS	Wash thrice	

**Table 4:** Primer sequences for angiogenic-related gene expression.

Gene	Sequences	Product Size	Accession
*GAPDH*	F: CAATGACCCCTTCATTGACC R: TTGATTTTGGAGGGATCTCG	160	NM_002046.7
*VEGFA*	F: TCACCAAGGCCAGCACATAG R: CCGGGATTTCTTGCGCTTTC	145	NM_001287044.2

**Table 5:** Statistical tests.

Parameter		Statistical Tests	Post Hoc
Pore size measurement		Kruskal–Wallis	Bonferroni-adjusted pairwise comparisons
Degradation rate		Friedman	Bonferroni-adjusted pairwise comparisons
Optimization of BMP-7 concentration		Kruskal–Wallis	Bonferroni-adjusted pairwise comparisons
Cytotoxicity test		Kruskal–Wallis	Bonferroni-adjusted pairwise comparisons
Viability of repopulated WJMSCs	Between group day 7	Mann–Whitney U	-
	Between group day 14, day 21	Independent *t*–test	-
	Within DHDP+WJMSC	Kruskal–Wallis	Bonferroni-adjusted pairwise comparisons
	Within DHDP+WJMSC+BMP-7	One-way ANOVA	Tukey
Angiogenic gene expression of WJMSCs		Mann–Whitney U	-

## Data Availability

The raw data supporting the conclusions of this article will be made available by the authors upon request.

## Abbreviations

The following abbreviations are used in this manuscript:

AA	Antibiotic-antimycotic mixture
ALK	Activin receptor-like kinases
bFGF	Basic fibroblast growth factor
BMP-7	Bone morphogenetic protein-7
DAPI	4′,6-diamidino-2-phenylindole
DHDP	Decellularized human dental pulp
DHDP+WJMSC	Wharton’s jelly mesenchymal stem cells repopulated within decellularized human dental pulp
DHDP+WJMSC+BMP-7	Wharton’s jelly mesenchymal stem cells repopulated within decellularized human dental pulp treated with bone morphogenetic protein-7
DMEM/F-12	Dulbecco’s Modified Eagle Medium nutrient mixture F12
DMEM/F-12 + 10% FBS	Dulbecco’s Modified Eagle Medium nutrient mixture F12 supplemented with 10% fetal bovine serum
ECM	Extracellular matrix
FBS	Fetal bovine serum
FESEM	Field emission scanning electron microscopy
GAG	Glycosaminoglycans
GAPDH	Glyceraldehyde-3-phosphate dehydrogenase
H&E	Hematoxylin and eosin
HDP	Human dental pulp
HLA-DR	Human leukocyte antigen DR isotype
IHC	Immunohistochemistry
MSC	Mesenchymal stem cells
mRNA	Messenger ribonucleic acid
OCT4	Octamer-binding transcription factor 4
PBS	Phosphate-buffered saline
PDGF-BB	Platelet-derived growth factor subunit bb
qRT-PCR	Quantitative reverse transcriptase polymerase chain reaction
RCT	Root canal treatment
RET	Regenerative endodontic therapy
SEM	Standard error of the mean
SDS	Sodium dodecyl sulfate
SOX2	Sex determining region y-box 2
VEGF-A	Vascular endothelial growth factor-A
VEGFR	Vascular endothelial growth factor receptor
WJMSCs	Wharton’s jelly mesenchymal stem cells
